# Adults with current asthma but not former asthma have higher all-cause and cardiovascular mortality: a population-based prospective cohort study

**DOI:** 10.1038/s41598-020-79264-4

**Published:** 2021-01-14

**Authors:** Xumei He, GeSheng Cheng, Lu He, Bing Liao, YaJuan Du, Xuegang Xie, Songlin Zhang, Gang Li, Yafeng Wang, YuShun Zhang

**Affiliations:** 1 Department of Structural Heart Disease, Xi’an Jiaotong University Medical College First Affiliated Hospital, Xi’an, 710061 Shaanxi Province China; 2 School of Nursing, Guangdong Pharmaceutical University, 283 Jianghai Avenue, Haizhu District, Guangzhou, China; 3 Department of Cardiac Surgery, Xi’an Children’s Hospital, Xi’an, 710061 Shaanxi Province China; 4 Department of Epidemiology and Biostatistics, School of Health Sciences, Wuhan University, Wuhan, China

**Keywords:** Asthma, Risk factors, Epidemiology

## Abstract

Higher mortality in asthmatics has been shown previously. However, evidence on different asthma phenotypes on long-term mortality risk is limited. The aim was to evaluate the impact of asthma phenotypes on mortality in general population. Data from the National Health and Nutrition Examination Survey from 2001–2002 to 2013–2014 linked mortality files through December 31, 2015, were used (N = 37,015). Cox proportional hazards regression was used to estimate the risk of all-cause and cause-specific mortality adjusting for sociodemographic characteristics, smoking, body mass index, and chronic conditions. During the mean follow-up time of 7.5 years, 4326 participants died from a variety of causes. Current asthma, but not former asthma was associated with increased all-cause mortality (current asthma: HR = 1.37; 95% CI 1.20–1.58; Former asthma: HR = 0.93; 95% CI 0.73–1.18); as well as mortality from cardiovascular disease (HR_Current_ = 1.41; 95% CI 1.08–1.85) and chronic lower respiratory diseases (HR_Current_ = 3.17; 95% CI 1.96–5.14). In addition, we found that the HR for cardiovascular disease (CVD) mortality was slightly greater in people with childhood-onset asthma than those with adult-onset asthma. The HR for chronic lower respiratory diseases (CLRD) mortality was greater in people with adult-onset asthma than those with childhood-onset asthma. However, the differences were not statistically significant. Our study suggested that current asthma but not former asthma was associated with increased all-cause, CLRD and CVD mortality. Future well-designed studies with larger sample are required to demonstrate the association and clarify the potential mechanisms involved.

## Introduction

Asthma is a common chronic inflammatory disease of the airways that is characterized by reversible airway obstruction and bronchospasm, which starts in childhood, becomes a major cause of morbidity in adults^1^. As main causes of disease burden as measured by disability-adjusted life years, asthma affected 1 to 18% of the populations in different countries and its prevalence of asthma continues to increase in the past decade^2,3^. In the United States, asthma affected 17% of children and 10.1% of adults^4,5^.

The previous studies have demonstrated the association between asthma and the higher risk of all-cause mortality^6–9^. However, the definition of asthma was inconsistent in different studies, and few studies distinguished difference between former asthma and current asthma on outcomes. In addition, asthma was demonstrated to associate with cardiovascular comorbidities and several studies showed that asthmatic adults have higher cardiovascular disease (CVD) mortality rates^10,11^. But the results based on longitudinal study were not consistent in general population. Evidence on the associations of asthma with the long-term cancer and other cause-specific mortality was also limited in the population-based cohort study. Moreover, early and late-onset asthma differ in regards to asthma triggers, sex, obesity, depression, and systemic inflammation^12^. The recent studies found that people with early-onset type 1 diabetes and type 2 diabetes have higher hazard ratios for most mortality^13^. What also remained unclear was whether asthma by disease onset (childhood vs adulthood) were differentially associated with all-cause mortality.

Therefore, we conducted this prospective cohort study to evaluate the association between asthma phenotypes and the risk of all-cause, CVD, cancer and cause-specific mortality. Subgroup analysis based on baseline characteristics and several sensitivity analysis were also performed in this study.

## Methods

### Study design and subjects

The data were from the National Health and Nutrition Examination Survey (NHANES). The previous study has describe the details of the NHANES design, methods and participants^14^. The seven waves of NHANES datasets from 2001–2002 and 2013–2014 were combined to increase statistical reliability, according to the NHANES analytic guidelines^15^.

The interviews of survey include demographic, dietary and health-related questions, including information regarding asthma status. The individuals were defined as having asthma if they have a positive response to the following question : ‘‘Has a doctor or other health professional ever told you that you have asthma?’’. If they have a positive response to the following question : ‘‘Do you still have asthma?’’, the individuals were defined as having current asthma. The individuals were defined as former asthma if they have a negative response to the question: ‘‘Do you still have asthma?’’. Of them, subjects with missing data on asthma status were excluded. We defined asthma as childhood and adulthood-onset subtypes according to the question: ‘‘How old were you when you were first told to have asthma? ’’, and the cut-off was 18 years old. The participants aged 20 years and older were included in our study. Subsequently, these participants were linked to death certificate data from the National Death Index through 31 December 2015 to determine the mortality status. A total of 37,015 participants aged 20 years and older were eligible for mortality follow-up. The detailed information about linkage of national center for health statistics survey data to the national death index was described elsewhere (https://www.cdc.gov/nchs/data-linkage/mortality-methods.htm). All survey participants provided informed consent prior to participating in the study. Institutional Review Board approval was not required since this study was based on secondary analyses of publicly available and deidentified data (https://www.cdc.gov/nchs/nhis/index.htm).

### Mortality outcomes

The all-cause and cause-specific mortality was ascertained according to the International Classification of Diseases, 10th Revision. CVD mortality was defined as I00 to I09, I11, I13, I20 to I51, or I60 to I69, and Cancer mortality was defined as C00–C97. The other cause-specific mortality included heart disease (I00–I09, I11, I13, I20–I51), Stroke (I60–I69), influenza and pneumonia (J09–J18), chronic lower respiratory diseases (CLRD) (J40–J47), diabetes mellitus (E10–E14), Alzheimer’s disease (G30), kidney disease (N00–N07, N17–N19, N25–N27) and accidents (unintentional injuries) (V01–X59, Y85–Y86). Because accidents deaths have no plausible links with asthma but do have similar confounding structure to mortality, and thus accidental deaths were used as a negative control outcome^16^.

### Covariates

Demographic characteristics included age, sex, race, education and income. Age was categorized as 20–65 and 66 years and older. Race were categorized as non-Hispanic black, Mexican American, non-Hispanic white and other. Education levels was divided into three groups: less than high school, high school graduate or equivalent, and more than high school. Income was assessed based on the annual family income relative to the federal poverty level (PIR). Participants were separated into 3 groups: PIR ≤ 1.3, 1.3 < PIR ≤ 3.5, and PIR ≥ 3.5. Body mass index was calculated as weight divided by height squared, which was divided into three groups: ≤ 25, 25.1–30 and ≥ 30. Smoking status was divided into three groups: never smokers, former smokers, or current smokers. Comorbid conditions included diabetes, arthritis, coronary heart disease, stroke, and cancer.

### Statistics analysis

We calculated the weighted percentage of asthma status across demographic characteristics, BMI, and comorbid conditions. The categorical variables were compared across asthma status using χ^2^ tests. The length of follow-up was defined as the interval from NHANES Interview date to the date of death or to the end of 2015 for those who were censored. We used Cox proportional hazards regression models to calculate hazard ratios (HRs) and 95% confidence intervals (CIs) for the association between asthma and all-cause CVD, cancer and cause-specific mortality. In model 1, we adjusted age and sex, and in model 2, we adjusted age, sex, race, education, income, smoking status, BMI, diabetes, arthritis, CHD, stroke and cancer. For all-cause mortality, CVD mortality and CLRD mortality, we calculated the HRs for early and late-onset current asthma. In addition, stratified analyses across covariates were also performed for all-cause mortality. To evaluate the robustness of the results, we also conducted sensitivity analyses through excluding participants with CHD, stroke and cancer at baseline or excluding participants with less than 2 years of follow-up. In addition, this study also calculated E-values to further assess the robustness of the presented results to residual confounding factors in a sensitivity analysis. The E-value estimates the minimum strength of an association between any unmeasured confounder, asthma (the exposure), and all-cause mortality (the outcome)^17^.

Take into account the complex sampling design, sampling weights were utilized in all analyses to account for unequal selection, probabilities and non-response, and the Taylor series method was used to estimate SE^18^. All analyses were conducted using STATA software, version 13.0 (Stata Corporation, College Station, TX, USA). The *P* values refer to two-tailed tests and statistical significance was set at *P* < 0.05.

## Results

This population cohort comprised a total of 37,015 individuals. Among them, 5.6% (n = 1896) and 8.0% (n = 2898) reported to have former and current asthma, respectively. Baseline characteristics of this study population according to asthma status were presented in Table 1. People with current asthma were female, Non-Hispanic White, and to have a lower income. Individuals with current asthma also had a higher prevalence of obesity, diabetes mellitus, CHD, stroke, cancer, and arthritis. Among current asthmatics, 1224 were adult-onset asthma, 1644 were adult-onset asthma and 30 had missing information on age when first had asthma.

**Table 1:** Baseline characteristics of the study participants according to asthma status.

Characteristics	No asthma (%) N = 32,221	Former asthma (%) N = 1896	Current asthma (%) N = 2898	Overall (%) N = 37,015
**Age (years)**				
20–65	24,603 (83.9)	1623 (90.5)	2283 (85.7)	28,509 (84.43)
66–	7618 (16.1)	273 (9.5)	615 (14.3)	8506 (15.57)
**Sex**				
Male	15,888 (49.2)	928 (48)	1049 (36.2)	17,865 (48.09)
Female	16,333 (50.8)	968 (52)	1849 (63.8)	19,150 (51.91)
**Race**				
Mexican American	5831 (8.7)	210 (5.5)	258 (4.1)	6299 (8.16)
Non-Hispanic White	14,910 (68.8)	931 (70.1)	1498 (72.3)	17,339 (69.14)
Non-Hispanic Black	6612 (11.1)	448 (12.4)	719 (13.3)	7779 (11.39)
Others	4868 (11.4)	307 (12)	423 (10.3)	5598 (11.32)
**Education level**				
< High school diploma	9015 (18.2)	381 (14.2)	778 (18)	10,174 (17.99)
High school graduate	7557 (24)	411 (21.2)	655 (22.7)	8623 (23.75)
> High school graduate	15,601 (57.6)	1104 (64.7)	1460 (59.2)	18,165 (58.16)
Missing	48 (0.1)	0 (0)	5 (0.1)	53 (0.1)
**Income**				
< 1.30	9039 (19.6)	578 (21.4)	1063 (27)	10,680 (20.27)
1.30–3.49	11,286 (33.9)	629 (33.1)	904 (31)	12,819 (33.59)
≥ 3.50	11,896 (46.6)	689 (45.5)	931 (42)	13,516 (46.14)
**BMI (kg/m**^**2**^**)**				
< 25	9790 (31.4)	559 (25.2)	666 (31.4)	11,015 (0)
25–30	10,939 (33.7)	601 (31.7)	782 (27.7)	12,322 (33.11)
> 30	10,744 (32.6)	700 (35.8)	1402 (45.5)	12,846 (33.77)
Missing	748 (1.8)	36 (1.1)	48 (1.6)	832 (1.73)
**Smoking status**				
Never smokers	17,496 (53.8)	975 (50.9)	1366 (48.8)	19,837 (53.27)
Former smokers	7939 (24.3)	449 (24.1)	770 (25.2)	9158 (24.34)
Current smokers	6758 (21.8)	472 (25)	761 (26)	7991 (22.34)
Missing	28 (0)	0 (0)	1 (0)	29 (0.044)
**Diabetes**				
No	28,451 (91.4)	1704 (93.1)	2387 (87.1)	32,542 (91.17)
Yes	3747 (8.5)	192 (6.9)	510 (12.8)	4449 (8.78)
Missing	23 (0.1)	0 (0)	1 (0)	24 (0.047)
**CHD**				
No	30,794 (96.6)	1820 (97)	2705 (94.4)	35,319 (96.4)
Yes	1291 (3.2)	67 (2.8)	176 (5.4)	1534 (3.31)
Missing	136 (0.3)	9 (0.3)	17 (0.3)	162 (0.29)
**Stroke**				
No	31,017 (97.4)	1837 (97.6)	2714 (94.7)	35,568 (97.16)
Yes	1158 (2.5)	57 (2.3)	182 (5.3)	1397 (2.74)
Missing	46 (0.1)	2 (0.1)	2 (0.1)	50 (0.093)
**Cancer**				
No	29,273 (90.9)	1729 (90.8)	2533 (87.2)	33,535 (90.61)
Yes	2914 (9)	166 (9)	359 (12.5)	3439 (9.28)
Missing	34 (0.1)	1 (0.1)	6 (0.4)	41 (0.11)
**Arthritis**				
No	24,092 (77)	1425 (77)	1655 (61.3)	27,172 (75.73)
Yes	8065 (22.9)	468 (22.9)	1236 (38.5)	9769 (24.11)
Missing	64 (0.2)	3 (0.1)	7 (0.2)	74 (0.16)

*BMI* body mass index, *CHD* coronary heart disease.

During a mean follow-up of 7.5 years (maximum duration was 15.1 years), there were 4326 deaths from all-cause, 950 deaths from cancer, 897 deaths from CVD, 733 deaths from heart disease, 164 deaths from stroke, 144 deaths from chronic lower respiratory disease, 86 deaths from diabetes, 64 deaths from influenza and pneumonia, 103 deaths from Alzheimer’s disease, 81 deaths from kidney disease, and 106 deaths from accidents.

### Asthma and the risk of all-cause mortality

**Figure 1:**
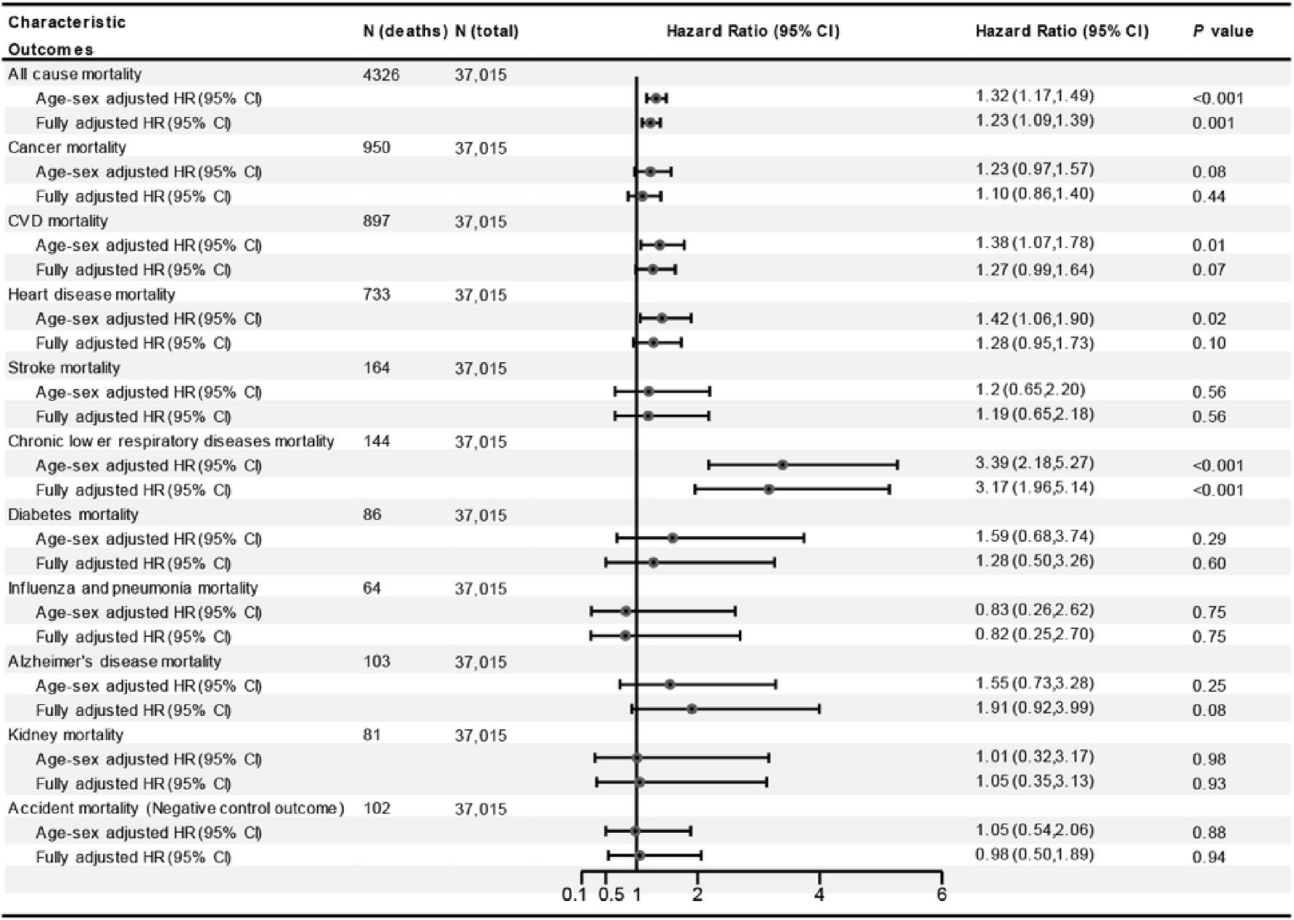
The association between asthma and all-cause, CVD and cancer and cause-specific mortality. *CI* confidence interval, *CVD* cardiovascular disease, *HR* hazard ratios. Multivariable models were adjusted for age, sex, race, education, income, smoking status, body mass index, diabetes, arthritis, coronary heart disease, stroke and cancer.

**Figure 2:**
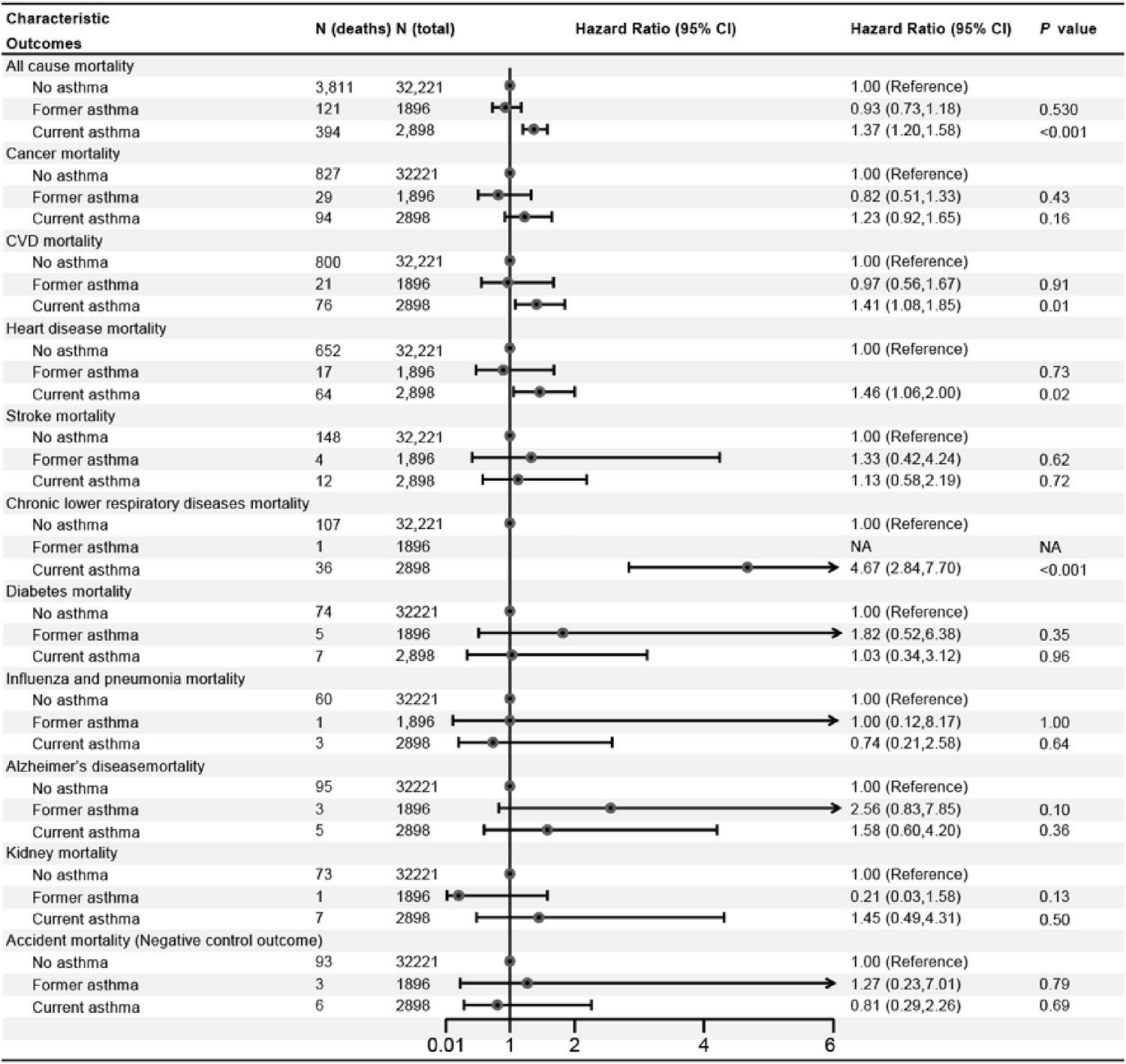
HRs for all-cause and cause-specific mortality among non-asthmatics, former asthmatics and current asthmatics. *CI* confidence interval, *CVD* cardiovascular disease, *HR* hazard ratios. Multivariable models were adjusted for age, sex, race, education, income, smoking status, body mass index, diabetes, arthritis, coronary heart disease, stroke and cancer.

**Figure 3:**
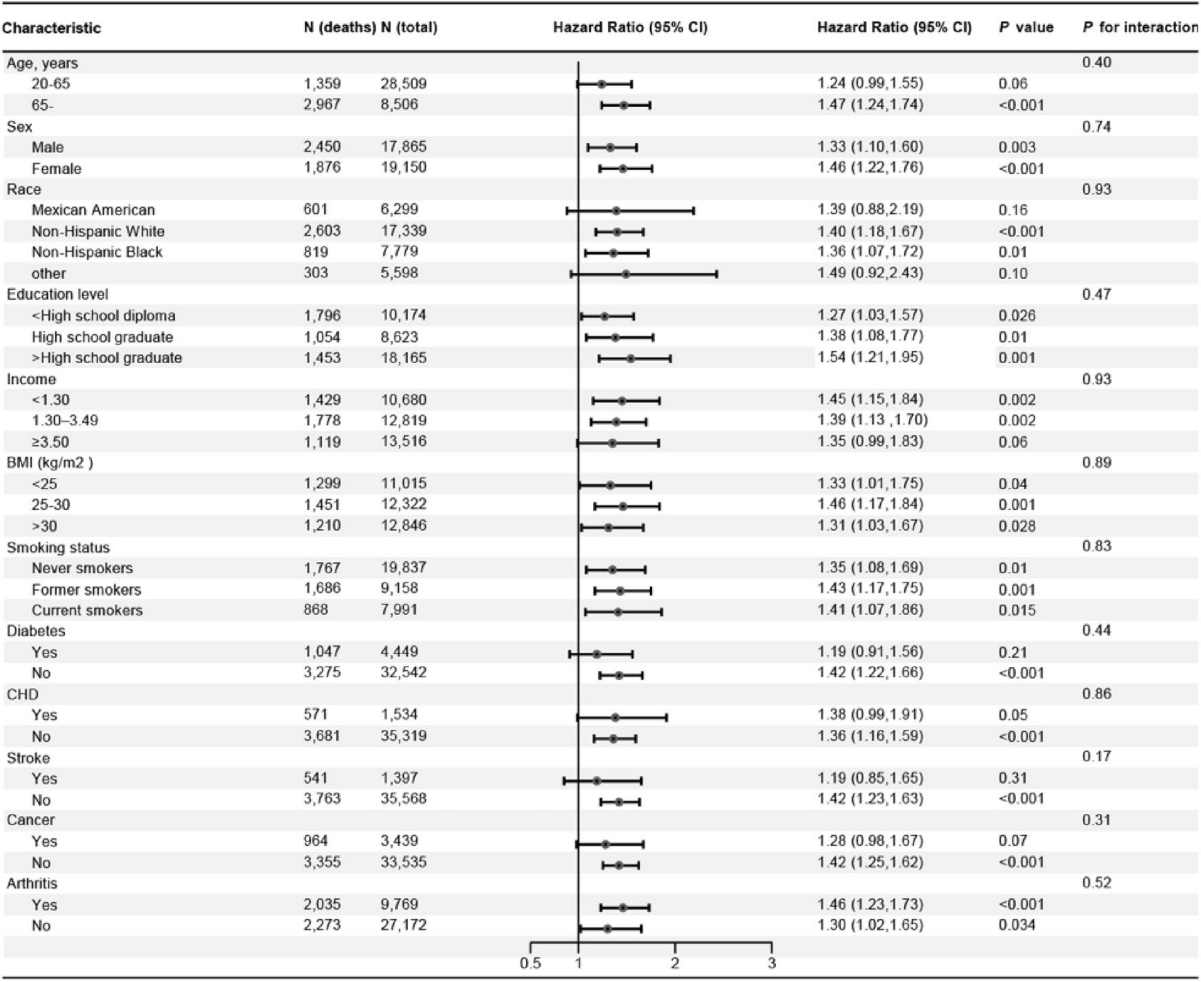
Subgroup analysis of the association of asthma with all-cause mortality (Current asthma vs. Non-asthma). *CI* confidence interval, *CVD* cardiovascular disease, *HR* hazard ratios. Multivariable models were adjusted for age, sex, race, education, income, smoking status, body mass index, diabetes, arthritis, coronary heart disease, stroke and cancer.

**Figure 4:**
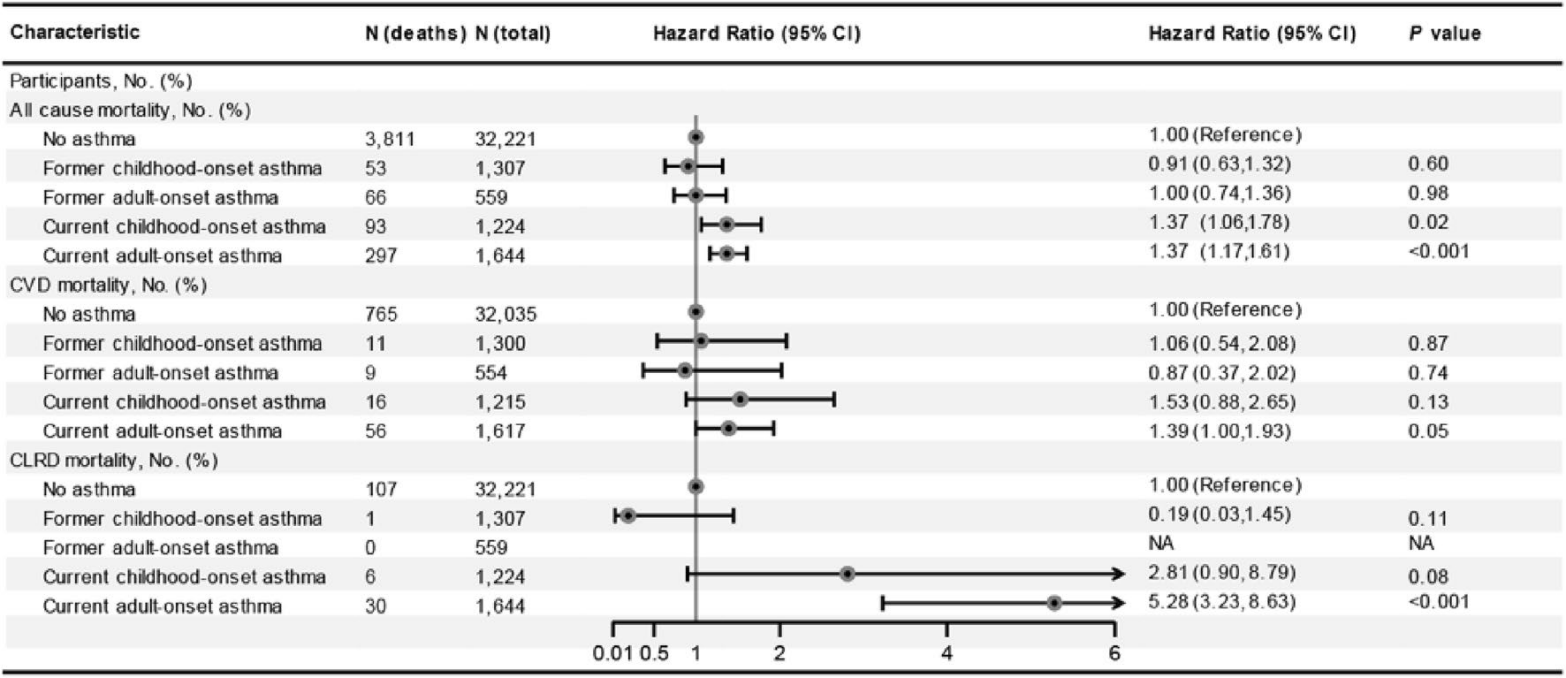
Risk of all-cause, CVD and CLRD mortality according to presence of asthma and its onset. *CI* confidence interval, *CLRD,* chronic lower respiratory diseases, *CVD* cardiovascular disease, *HR* hazard ratios. Multivariable models were adjusted for age, sex, race, education, income, smoking status, body mass index, diabetes, arthritis, coronary heart disease, stroke and cancer.

In the age and sex-adjusted model, individuals with asthma had a 32% (HR = 1.32; 95% CI 1.17–1.49) increased risk of all-cause mortality (Fig. 1). In the multi-adjusted model, although the association attenuated, asthma was still associated with increased risk of all-cause mortality (HR = 1.23; 95% CI 1.09–1.39; Fig. 1). Subsequently, asthma was divided into former and current asthma, and the results showed that current asthma was associated with 37% increased risk of all-cause mortality (HR = 1.37; 95% CI 1.20–1.58; Fig. 2). However, there was no association between former asthma and mortality (HR = 0.93; 95% CI 0.73–1.18). Subgroup analyses showed that almost all findings remained consistent for the associations observed in the main effects models (all pinteraction > 0.05; Fig. 3), although the adjusted HRs were higher in participants who were older, female, higher education, and with arthritis (Fig. 3). In addition, compared to non-asthma, current adult-onset asthma and childhood-onset asthma have a similar all-cause mortality risk (adulthood asthma: HR = 1.37; 95% CI 1.06–1.78; Childhood asthma: HR = 1.37; 95% CI 1.17–1.61; Fig. 4).

### Asthma and the risk of CVD, cancer and cause-specific mortality

In the multi-adjusted model, asthma was associated with higher chronic lower respiratory diseases mortality risk (HR = 3.17; 95% CI 1.96–5.14; Fig. 1). There were no associations between asthma and CVD, cancer, diabetes, influenza and pneumonia, Alzheimer’s disease, and kidney disease mortality risk (Fig. 1). However, current asthma was associated with 41% increased risk of CVD mortality (HR = 1.41; 95% CI 1.08–1.85; Fig. 2) and former asthma was not still associated with this risk (HR = 0.97; 95% CI 0.56–1.67). In addition, we found that the HR for CVD mortality was slightly greater but not significant in people with childhood-onset asthma than those with adult-onset asthma. The HR for CLRD mortality was greater in people with adult-onset asthma than those with childhood-onset asthma (Fig. 4).

### Sensitivity analysis

**Figure 5:**
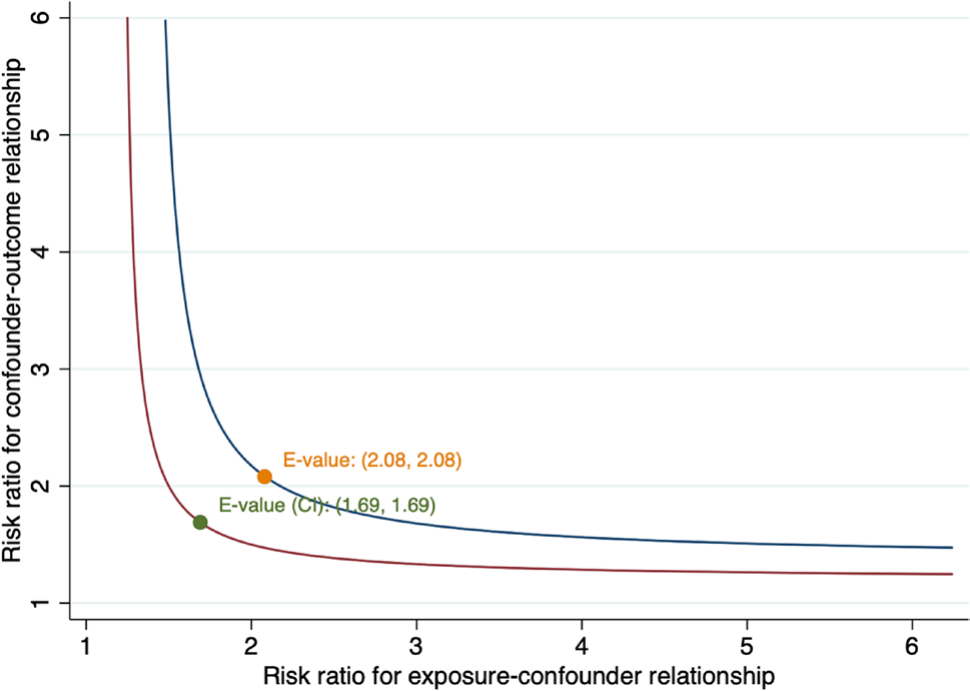
Value of the joint minimum strength of association on the risk ratio scale that an unmeasured confounder must have with the exposure and the outcome to fully explain away an observed current asthma—all-cause mortality HR.

When the analysis excluded individuals with a history of CHD, stroke, and cancer at baseline or participants with less than 2 years of follow-up, the results were generally similar to those of the main analysis (Table 2). In addition, no evidence of association was observed for asthma status and the negative control outcomes, which indicated the robustness of our results. Moreover, further E-value sensitivity analysis also conducted to evaluate the robustness of the association to unmeasured confounding. The observed HRs of 1.37 for the outcome (all-cause mortality) associated with exposure (current asthma) could be explained by an unmeasured confounder, which was associated with current asthma. Furthermore, current asthma and all-cause mortality had HRs of at least 2.08 (Fig. 5) beyond the measured confounders, but not by weaker confounding (The corresponding CI is at least 1.69).

**Table 2:** Sensitivity analysis for association between asthma and all-cause mortality risk.

Outcomes	Participants	Deaths	HR (95%CI)	*P* value
**Excluding participants with CHD, stroke and cancer**				
No asthma	32,170	3776	1.00 (Reference)	
Former asthma	1895	121	0.93 (0.73,1.18)	0.50
Current asthma	2888	390	1.38 (1.21,1.59)	< 0.001
**Excluding participants with ≤ 2 years of follow-up**				
No asthma	29,371	3092	1.00 (Reference)	
Former asthma	1710	100	1.01 (0.77,1.32)	0.96
Current asthma	2551	293	1.31 (1.13,1.52)	< 0.001

*CI* confidence interval, *CLRD* chronic lower respiratory diseases, *CHD* coronary heart disease, *HR* hazard ratio, *NA* not available.

## Discussion

The aim of this study was to evaluate the impact of 'asthma' on mortality risk in the general population, both in terms of current adult-onset and childhood-onset asthma. We found that current asthma but not former asthma was significantly associated with the increased risk of CVD, chronic lower respiratory diseases and all-cause mortality. However, individuals with current asthma had significant higher mortality risk from heart disease but not stroke. Further subgroup analyses showed that estimate of HR for total deaths was slightly higher in women than in men and in older individuals than younger individuals, but they were not statistically significant.

Several large longitudinal studies and meta-analyses have provided strong evidence about asthma for risk of cardiovascular disease or all-cause mortality^7,10,19,20^. The meta-analysis including ten studies of 406, 426 participants showed that compared to individuals without asthma, asthma was associated with an 36% and 33% higher risk of total deaths (RR 1.36; 95% CI 1.01–1.83) and CVD events (RR 1.33; 95% CI 1.15–1.53), but with significant heterogeneity between studies^11^. Another large prospective cohort study also showed that those with asthma had an increased all-cause mortality risk^21^. Although our results also found higher all-cause mortality risk in those with asthma, we did not observe the significant association between asthma and CVD mortality risk. The asthma definition from most of these previous studies was according to only one question related to asthma history (Yes/No), which limited them to evaluate the current and former asthma and mortality risk^10,11^. After using a stricter definition of asthma, our study showed an increased risk of deaths from all-cause and CVD in individuals with current, but not former asthma. In consistent with our finding, recent another cohort study also observed that active asthma was associated with a 32% increased CVD mortality risk (HR 1.32, 95% CI 1.08–1.62), but there was no significant relationship between non-active asthma and this adverse cardiovascular consequences (HR 0.96, 95% CI 0.77–1.62), indicating that the true association between asthma and mortality risk might be underestimated and in the future more studies using the stricter definition dividing asthma into current and former were needed to demonstrate this finding^11^.

Although the pathogenesis that asthma may influence CVD remains elusive, several potential mechanisms have been suggested to explain the cause the observed association in risk of cardiovascular disease associated with asthma^21–23^.

One possibility was that chronic airway inflammation might contribute to low-grade systemic inflammation in asthmatic patients and could influence later risk of CVD^10,24,25^. Previous studies also suggested that compared with persons who never had asthma, asthmatic patients had elevated has inflammatory biomarkers such as C-reactive protein, interleukin-1β, tumor necrosis factor-alpha, interleukin-6, interleukin-8, and fibrinogen which could increase progress of atherogenesis and then lead to the occurrence of CVD events^24,26^.

Besides, lung function impairment also played an important role in the association of asthma with CVD risk^27,28^. Long-term airway remodeling in those with asthma, could induce irreversible airway obstruction and lead to lung function impairment. A decline in lung function with lower pre-bronchodilator forced expiratory volume in 1 s (FEV1) was demonstrated to be associated with CVD risk^27,28^. In addition, another hypothesis was that asthma medication use, such as oral or inhale glucocorticoids and beta-adrenoceptor agonists might have the cardiotoxic effects which could increase subsequent CVD risk^29,30^. Moreover, asthma and cardiovascular disease shared many common risk factors such as obesity, air pollutants, smoking, psychological stress, or physical inactivity, which could partly explain the relationship between asthma and CVD risk^11,31,32^. Previous study showed that patients current asthma had a significant elevated C-reactive protein concentration than those with former asthma or never asthma, but there was not significant difference in C-reactive protein concentration among those with former asthma and non-asthma which supported our finding that current asthma not former asthma was associated with increased CVD, chronic lower respiratory diseases and all-cause mortality^26^. Asthma was such a heterogenous disease, adult-onset and childhood-onset asthma appeared to run a different course and had different characteristics^33^. Childhood-onset asthma was mild and remission was common^33^. Our study showed that HR for CVD mortality was a slightly higher among individuals with current childhood-onset asthma than those with current adulthood-onset asthma, and individuals with adulthood-onset asthma had a greater HRs for CLRD mortality than those with childhood-onset asthma. But their CIs were wider and overlapped which might partly induced by probably small sample size.

### Strengths and limitations

The present study has some strengths. Firstly, among individuals with asthma, this study was able to separate patients with asthma by disease status (current vs former), and disease onset (ie, childhood vs adulthood). Secondly, a serial of sensitivity analysis including using a negative control outcome and calculating E-value support the robustness of the main results. Despite the numerous strengths in this study, there are also several potential limitations. Firstly, due to the nature of observational study, the described associations do not infer causation. In addition, the sample size for our dataset was relatively small and future well-designed study with larger sample are required to evaluate the associations of asthma with mortality risk. Secondly, asthma status were self-reported which might be subject to recall and reporting bias. Although lack of clinical or laboratory data limited this study to validate asthma status, self-reported asthma commonly used in population-based studies had been evaluated and displays acceptable sensitivity and specificity^34,35^. Thirdly, although in our analyses, a number of potential confounders were adjusted, we did not completely rule out a possibility of residual and unmeasured confounding such as chronic bronchitis or emphysema. The chronic respiratory diseases frequently co-occured or were misclassified in clinical practice, especially for misclassification with other respiratory disease typically occurring in older adults who might be smokers. Therefore, estimates in our study might be overestimated due to lack of controlling for these variables. Fourthly, lack of laboratory data such as systemic inflammation biomarkers limited us to further explore the potential biological mechanisms involved.

## Conclusion

Our study suggested that current asthma but not former asthma was associated with increased all-cause, CLRD and CVD mortality. Age at disease onset may be an important determinant of CLRD and CVD mortality among current asthmatics. Future well-designed studies with larger sample are required to demonstrate the association and clarify the potential mechanisms involved.

## Data Availability

The NHANES data are available from https://www.cdc.gov/nchs/nhanes/.
